# Predicting head injury prognosis using clinical scores (GCS and GCS-P score)

**DOI:** 10.6026/973206300222118

**Published:** 2026-04-30

**Authors:** Atul Kumar, Shivani Sinha, Bhavesh Khandelwal, Neelam R Charles

**Affiliations:** 1 Department of Surgery, Government Medical College, Ratlam, Madhya Pradesh, India; 2 Department of Microbiology, Government Medical College, Ratlam, Madhya Pradesh, India

**Keywords:** Glasgow coma scale (GCS), head injuries, prognosis

## Abstract

The Glasgow Coma Scale (GCS) and the Glasgow Coma Scale-Pupils (GCS-P) are critical tools for predicting prognosis in patients with head injury. Therefore, it is of interest to evaluate the comparative efficacy of both scales in determining mortality and functional recovery following traumatic brain injury. By integrating the Pupil Reactivity Score (PRS), the GCS-P addresses the "floor effect" of the traditional GCS and provides a more granular assessment of brainstem compromise. Our analysis demonstrates that the GCS-P offers superior predictive accuracy and a more linear correlation with patient outcomes than the GCS alone. Thus, we show that adopting the GCS-P enhances early clinical triage and provides a more robust framework for neurological prognostic modeling.

## Background:

Assessment of a patient's clinical condition and how it may change and react to this at earliest moment is the cornerstone in the management of patients with head injury. The Glasgow Coma Scale (GCS) is widely used for this purpose which was developed by Professor Bryan Jennett and Sir Graham Teasdale in the Institute of Neurological Sciences in Glasgow [1]. It is a scale that has been accepted worldwide and used by, not only doctors, but nurses and emergency staff as well. Even though the Glasgow Coma Scale has been accepted as the gold standard in neurological assessment of patients, it has come down under scrutiny and many authors have claimed weaknesses in the scoring system as this scale dose not measure the focal indices like brainstem functions [2]. This includes its inability to predict a patient outcome and having variation in its reading amongst assessing individuals. The Glasgow Coma Scale is used to assess 3 aspects of a patient's responsiveness (eye, verbal and motor responses) [3]. Each of these aspects contains information about prognosis. Brain damage, which is the most important feature in distinguishes head injuries of differing severities and in monitoring patient progress and estimating prognosis [4]. The GCS score, together with information about pupil reaction, conveys to the physician most of the clinical predictive information in head-injured patients [5]. Therefore, it is of interest to determine GCS and GCS-P Score and analyze these two scores in the determining the prognosis of patient with head injury as per Glasgow Coma Scale.

## Methodology:

This observational study was conducted from March 2022 - February 2023 in laxmi Narayan Pandey Govt. Medical College Ratlam. All the cases of head injury attending OPD or admitted in casualty and surgical department in Government Medical College and allied hospitals, Ratlam, Madhya Pradesh were included in this study. Patients were managed as per ATLS guidelines. Demographic characterization of the patients, mode of injury, nature of head injury and other relevant histories were recorded. Relevant blood investigations, X-ray, USG abdomen/ thorax and CT scan head was done as per patient's clinical condition. Patients with GCS 13 or less, Age 16 years and above with Closed and Open Head injuries were included in the study. Patients with GCS 14 or above and having comorbidities such as renal, liver failure were excluded from the study. Initial Assessment of the patient will be done by Glasgow Coma Scale and GCS score and GCS-P Score will be recorded and will be reassessed and recorded as per clinical condition of the patient. The minimum score will be taken for calculation as sown in Table 1 (see PDF). GCS-P Score will be obtained by subtracting the pupil reactivity score (PRS) from the GCS total score.

## Results:

In this study, we have analyzed each component of the Glasgow Coma Scale and compared it with the Glasgow Coma Scale for sample size of thirty patients with head injury who came to Dr Laxmi Narayan Pandey Government medical college, Ratlam, Madhya Pradesh came in casualty or admitted in surgery department march 2022 to end of Feb 2023 All the head injury patients underwent resuscitation and CT brain and admitted in the surgical intensive care unit. Consents from patient's close relatives were obtained and, aside from the initial GCS score, the patients were assessed with regard to sex and mode of injury which was interpreted through Chi-square analyses. To interpret the results, it should be known that the scores of each component of GCS was divided into those with no response ('1') and those with response ('2' and above). As was Glasgow Outcome scale. A scale with five categories, was pooled into two categories; good recovery/ moderately disabled (favorable outcome) and severely disabled/vegetative/ death (unfavorable outcome). Upon admission, Glasgow coma score was taken routinely. The patient was stabilized by ensuring adequate oxygenation and blood flow. Once stabilized, the patient was shifted to the neurosurgery ICU and first GCS score was taken. Out of the 164 patients who participated in this study, 44 patients were of severe head injury status according to GCS scoring system i.e., GCS score of 8 or less. The remaining 120 patients were of moderate brain injury (GCS of 9-13). The mean age of the sample of patients was 37.4 years old. The percentage of patients with favorable outcome' as GOS was 81.7 % (134/164) and with unfavorable outcome was 19.3 % (30/164). Patients with GCS less than 5 had mortality rate of 89.4 % (17/19). On collection of cases, it was noted that the majority of patients who had come to the emergency department with head trauma where of male gender. A total of only 50 patients out of 164 patients were female. Majority of patients who sustained trauma by road traffic accidents was the major cause of head injury in this study followed by fall from height and assault. 19.16 % of the patients with RTA had unfavorable outcome while 15 % and 16.6 % of patients OD fall and assault had unfavorable outcome respectively as shown in Table 2 (see PDF). In this study mode of injury did not show to be of significance with regard to the final Glasgow Coma Scale. In this study all patients with GCS less than 5 with bilateral unreactive pupil were not able to survive while the survival rate is 25 % and 50 % in patient with one and both reactive pupil. Favorable outcome was observed in patient with both reactive pupils regardless of GCS score. 97 % in patients with GCS 9 to 13, 88.9 % in GCS 5to 8 and 50 % with GCS less than 5 as compared to 80 %, 60% and 0 % in patients bilateral non-reactive pupil and 89.9 %, 80 % and 25 % in patients with one reactive pupil as shown in Table 3 (see PDF).

## Discussion:

Traumatic brain injury (TBI) remains a significant cause of morbidity and mortality worldwide, necessitating reliable tools for early prognostication [6]. The Glasgow Coma Scale (GCS) has long served as the gold standard for assessing consciousness and neurological status in head injury patients [1]. However, it's limitations- particularly in severe TBI-have prompted the development of enhanced scoring systems such as the Glasgow Coma Scale-Pupil (GCS-P), which incorporates pupillary reactivity to improve predictive accuracy [3]. In our study, both GCS and GCS-P scores were evaluated for their ability to predict short- and long-term outcomes in patients with head injury [2]. The results demonstrated that while GCS remains a robust tool for initial assessment, GCS-P offers superior prognostic value, especially in cases of severe TBI [5]. The inclusion of pupillary response in GCS-P allows for a more nuanced assessment of brainstem function-an area not addressed by the traditional GCS [7]. Pupillary non- reactivity has been independently associated with poor outcomes and its integration into the scoring system enhances the clinician's ability to stratify patients based on risk [8]. The enhanced predictive power of GCS-P has several clinical implications. First, it facilitates more accurate triage decisions in emergency settings, guiding the need for advanced imaging, neurosurgical consultation and ICU admission [9]. Second, it supports informed discussions with families regarding prognosis and potential outcomes [10]. Third, it may serve as a valuable tool in resource-limited settings where access to neuroimaging is constrained, allowing for rapid bedside assessment of injury severity. Moreover, GCS-P can be integrated into trauma registries and predictive models to improve outcome tracking and quality of care metrics.

## Conclusion:

We show the importance of incorporating pupillary reactivity into neurological assessment through the GCS-P score. While GCS remains a cornerstone of head injury evaluation, GCS-P provides a more comprehensive and accurate prediction of outcomes, particularly in severe cases. Thus, its adoption in clinical practice can improve decision-making, optimize resource allocation and ultimately enhance patient care.

## Limitations:

Despite its advantages, the GCS-P score is not without limitations. Inter-observer variability in assessing pupillary response may affect scoring accuracy, particularly in pre-hospital or low-resource environments. Additionally, factors such as ocular trauma, pharmacologic agents (e.g., atropine) and pre-existing neurological conditions can confound pupillary assessment. Our study was conducted at a single tertiary care center with a modest sample size, which may limit the generalizability of the findings. Future multicentric studies with larger cohorts and standardized assessment protocols are needed to validate the utility of GCS-P across diverse populations and clinical settings.

## References

[1] Bertotti MM (2023). Arq Neuropsiquiatr..

[2] Mahajan C (2023). Brain Inj..

[3] Ahmadi S (2023). Eur J Trauma Emerg Surg..

[4] Jain H (2025). J Trauma Inj..

[5] Singh RD (2022). Trials..

[6] Zhou J (2026). BMJ Open..

[7] Vreeburg RJG (2025). J Neurotrauma..

[8] Pisano F, Bilotta F (2024). J Head Trauma Rehabil..

[9] Menon DK (2025). J Neurotrauma..

[10] Nelson LD (2025). J Neurotrauma..

